# Protection of Stem Cell-Derived Lymphocytes in a Primate AIDS Gene Therapy Model after In Vivo Selection

**DOI:** 10.1371/journal.pone.0007693

**Published:** 2009-11-02

**Authors:** Grant D. Trobridge, Robert A. Wu, Brian C. Beard, Sum Ying Chiu, Nina M. Muñoz, Dorothee von Laer, John J. Rossi, Hans-Peter Kiem

**Affiliations:** 1 Clinical Research Division, Fred Hutchinson Cancer Research Center, Seattle, Washington, United States of America; 2 Department of Medicine, University of Washington, Seattle, Washington, United States of America; 3 Department of Pathology, University of Washington, Seattle, Washington, United States of America; 4 Georg-Speyer-Haus, Institute for Biomedical Research, Frankfurt, Germany; 5 Department of Molecular Biology, Beckman Research Institute of City of Hope, Duarte, California, United States of America; University of California San Francisco, United States of America

## Abstract

**Background:**

There is currently no effective AIDS vaccine, emphasizing the importance of developing alternative therapies. Recently, a patient was successfully transplanted with allogeneic, naturally resistant CCR5-negative (CCR5Δ32) cells, setting the stage for transplantation of naturally resistant, or genetically modified stem cells as a viable therapy for AIDS. Hematopoietic stem cell (HSC) gene therapy using vectors that express various anti-HIV transgenes has also been attempted in clinical trials, but inefficient gene transfer in these studies has severely limited the potential of this approach. Here we evaluated HSC gene transfer of an anti-HIV vector in the pigtailed macaque (*Macaca nemestrina*) model, which closely models human transplantation.

**Methods and Findings:**

We used lentiviral vectors that inhibited both HIV-1 and simian immunodeficiency virus (SIV)/HIV-1 (SHIV) chimera virus infection, and also expressed a P140K mutant methylguanine methyltransferase (MGMT) transgene to select gene-modified cells by adding chemotherapy drugs. Following transplantation and MGMT-mediated selection we demonstrated transgene expression in over 7% of stem-cell derived lymphocytes. The high marking levels allowed us to demonstrate protection from SHIV in lymphocytes derived from gene-modified macaque long-term repopulating cells that expressed an HIV-1 fusion inhibitor. We observed a statistically significant 4-fold increase of gene-modified cells after challenge of lymphocytes from one macaque that received stem cells transduced with an anti-HIV vector (p<0.02, Student's t-test), but not in lymphocytes from a macaque that received a control vector. We also established a competitive repopulation assay in a second macaque for preclinical testing of promising anti-HIV vectors. The vectors we used were HIV-based and thus efficiently transduce human cells, and the transgenes we used target HIV-1 genes that are also in SHIV, so our findings can be rapidly translated to the clinic.

**Conclusions:**

Here we demonstrate the ability to select protected HSC-derived lymphocytes in vivo in a clinically relevant nonhuman primate model of HIV/SHIV infection. This approach can now be evaluated in human clinical trials in AIDS lymphoma patients. In this patient setting, chemotherapy would not only kill malignant cells, but would also increase the number of MGMTP140K-expressing HIV-resistant cells. This approach should allow for high levels of HIV-protected cells in AIDS patients to evaluate AIDS gene therapy.

## Introduction

AIDS continues to be a serious health problem with an estimated 33 million people infected worldwide [1]. Highly active antiretroviral therapy reduces viral loads and morbidity and mortality from AIDS [2], but the side effects can be severe [3] and the emergence of drug resistant HIV strains is a problem. Despite substantial efforts to develop a vaccine [4], there is still no cure [5] and alternative therapies need to be explored. One approach to cure AIDS has been by transplantation with genetically-modified T cells that inhibit HIV replication. Several synthetic genes have been developed which can inhibit infection or replication of HIV-1 using a gene therapy approach [6]–[8]. Clinical trials have been performed using T cell gene transfer that show efficacy [9], but T cells have a limited life span in vivo which will likely limit the utility of this approach. The ability of hematopoietic stem cells (HSCs) to reconstitute the entire hematopoietic system, including the T cell repertoire, macrophages, dendritic cells and microglial cells, suggests they are ideal targets for AIDS gene therapy. The recent report that allogeneic HSC transplantation with naturally-resistant CCR5-negative HSCs cured a patient with AIDS [10] lends support to this approach as a viable therapeutic option, at least until an effective vaccine can be developed.

However, the inability to efficiently deliver anti-HIV transgenes to HSCs has been a major roadblock in clinical trials. In a phase I clinical trial using bone marrow-derived CD34^+^ cells transduced with a Moloney leukemia virus vector containing a Rev response element (RRE) decoy short-term marking rates of 0.003–0.01% and long-term marking rates below 0.001% were obtained [11]. In another study where mobilized CD34^+^ cells were used as targets, the average long-term marking rates with a Moloney leukemia virus vector containing a ribozyme were 0.01% to 0.001% [12]. More recently, a large randomized, double-blind, phase 2 gene transfer clinical trial was conducted in 74 HIV-1-infected adults who received a tat-vpr-specific anti-HIV ribozyme (OZ1) or placebo delivered in autologous CD34^+^ hematopoietic progenitor cells. In this trial there were no OZ1-related adverse events but there was also no statistically significant difference in viral load between the OZ1 and placebo group at the primary end point. Again, marking was very low in this study and vector DNA did not reach the quantifiable range of the assay (0.38% of cells analyzed) in any blood cell sample at any time point. The authors concluded that the approach was promising but that improvements to increase engraftment were needed [13].

We previously established conditions for efficient transduction of pigtailed macaque (*Macaca nemestrina*) long-term repopulating cells using vesicular stomatitis virus glycoprotein (VSV-G) pseudotyped HIV-based lentiviral vectors [14]. Stable, long-term, polyclonal high-level gene marking was observed using relatively low multiplicities of infection (MOI)s, and transgene expression was detected in all lineages. The high levels of gene transfer observed are likely due to the absence of a functional TRIM5α in pigtailed macaques [15]. Pigtailed macaques can be infected with either SHIV or SIV strains. SHIV is a chimera that contains the HIV-1 *env*, *rev*, *tat*, and *vpu* genes on a background of SIVmac, and infects macaques causing simian AIDS [16]. Infection of pigtailed macaques with SHIV thus offers an excellent preclinical model to evaluate AIDS HSC gene therapy using lentiviral vectors.

Here we developed and evaluated lentiviral-based anti-HIV vectors in the pigtailed macaque (*Macaca nemestrina*) nonhuman primate model. The lentiviral vectors we used also contain the P140K version of the O^6^-methylguanine-DNA methyltransferase (MGMT) gene [17] to increase marking after transplantation. The P140K MGMT mutant confers resistance to methylating agents such as temozolomide as well as to nitrosoureas such as BCNU (bis-chloronitrosourea), and allows for efficient selection at the stem cell level in large animal models [18]–[20]. The MGMT protein is expressed in normal human tissues, so in order to enhance selection, a potent inhibitor, O^6^-benzylguanine (O6BG) is used to inactivate endogenous MGMT. The mutant P140K gene is resistant to O6BG [21], [22], so that an alkylating agent such as BCNU or temozolomide used in conjunction with O6BG allows for efficient selection of transduced HSCs. We evaluated marking and MGMT-mediated selection of macaque repopulating cells transduced with an anti-HIV lentiviral vector that expresses a membrane-bound C46 fusion inhibitor [23]. A competitive repopulation assay with enhanced green and yellow fluorescent protein (EGFP/EYFP) markers allowed us to evaluate marking of a combinatorial anti-HIV vector. The macaque competitive repopulation model we have developed here allows for a facile and quantitative assessment of the engraftment potential and also efficacy of vectors proposed for AIDS gene therapy clinical trials.

Importantly, this approach can be rapidly translated to the clinic because the vectors we describe here target HIV-1, and transplantation in this primate model closely models human transplantation. Additionally, the MGMT selection strategy should be particularly effective for patients with AIDS lymphoma who will require an HSC transplant and may also be treated with the chemotherapy agent, BCNU which could increase marking of the MGMTP140K anti-HIV vector.

## Materials and Methods

### Ethics Statement

Healthy juvenile pigtailed macaques (*Macaca nemestrina*) were housed at the University of Washington National Primate Research Center under conditions approved by the American Association for Accreditation of Laboratory Animal Care. Study protocols were approved by the Fred Hutchinson Cancer Research Center (FHCRC) Institutional Review Board and the FHCRC Institutional Animal Care and Use Committee. All animals received myeloablative total-body irradiation and received recombinant human granulocyte colony stimulating factor (rhG-CSF) and standard supportive care after transplantation. Animals are monitored closely and animal welfare is assessed on a daily basis, and if necessary several times a day. This includes veterinary examinations to make sure animals are not suffering. If animals experience pain they receive pain medications. If pain can not be relieved, or if veterinary examination reveals signs of suffering that cannot be relieved by analgesics, antiemetics, or antibiotic therapy, animals are euthanized.

### Lentiviral anti-HIV MGMT vectors

The lentiviral vector plasmid pRSC-SMPGW contains a spleen focus forming virus (SFFV) long terminal repeat (LTR)-derived promoter-driven MGMT transgene and a phosphoglycerate kinase (PGK) promoter-driven EGFP reporter gene and has already been described [14]. The C46 anti-HIV transgene that encodes a partial ORF from the HIV envelope protein transmembrane subunit spanning from amino acids 628 to 673 of gp41 [23] was inserted 3′ to the SFFV promoter using standard cloning techniques and an internal ribosome entry site (IRES) derived from the encephalomyocarditis virus was placed 3′ to the C46 transgene and upstream of the MGMT transgene so that the C46 is expressed from SFFV and MGMT is expressed from an IRES to create the resulting vector plasmid pRSC-SC46-IMPGW. The U6 Pol III promoter-driven site I shRNA targeting *tat* and *rev* [24] was inserted 5′ to the SFFV promoter of the pRSC-SC46-IMPGW plasmid to create the vector plasmid pRSC-UsI-SC46-IMPGW. HIV-based vectors were pseudotyped with VSV-G envelope and produced by transient transfection of 293T cells and concentrated 100-fold as previously described [25].

### Cell lines

Human 174xCEM cells [26], CEM.NKR-CCR5 cells [27], HeLa-CD4-LTR-β-gal cells [28] and MAGI-CCR-5 cells [29] were obtained through the AIDS Research and Reference Reagent Program, Division of AIDS, NIAID, from Dr. Peter Cresswell, Dr. Alexandra Trkola, Dr. Michael Emerman and Dr. Julie Overbaugh, respectively. 174xCEM and CEM.NKR-CCR5 cells were cultured in Iscove's Modified Dulbecco's Medium, and human embryonic kidney 293 cells, HeLa-CD4-LTR-β-gal and MAGI-CCR-5 cells were cultured in Dulbecco's modified Eagle's medium. All cell lines were supplemented with 10% heat-inactivated FBS, 100 U/ml of penicillin, and 100 µg/ml of streptomycin and were incubated at 37°C in a 5% CO_2_ atmosphere.

### Preparation of HIV and SHIV challenge virus

The following viruses, infectious virus plasmids, and cell lines were obtained through the AIDS Research and Reference Reagent Program from the following investigators. SHIV KU-1 was obtained from Dr. Opendra Narayan and Dr. Sanjay Joag. HIV-1 89.6 [30] was obtained from Dr. Ronald Collman. Virus preparations were titered using HeLa-CD4-LTR-β-gal cells as previously described [28].

### Construction of a mutant lentiviral rev helper plasmid for efficient production of anti-HIV vectors containing *rev*-specific shRNAs

The lentiviral helper plasmid pCMVdeltaR8.74 was first digested with *EcoRI* and *Not*I, then klenow-filled and ligated to create a smaller 5.3 kb plasmid containing the *rev* target site. This plasmid was then PCR amplified using two 5′ phosphorylated primers with synonymous mutations in *rev* (5′-GACGAGGAACTCATCAGAACAGTCAGACT-3′ and 5′- CGACAGCGGCGAAGAAGGACGGTAT-3′) that formed a junction within the target rev sequence using the high fidelity Phusion enzyme (NEB, Ipswitch, MA) following the manufacturer's recommendations. Following five cycles of PCR with a lower annealing temperature of 60°C, the annealing temperature was then raised to 65 for an additional 25 cycles. The resulting linear PCR product was ligated and transformed into *E. coli*. From this plasmid a 2.2 kb NheI to SalI fragment was shuttled back into pCMVdeltaR8.74 creating the plasmid pCMVdeltaR8.74-sIM. The mutated *rev* target site of pCMVdeltaR8.74-sIM was sequenced to confirm the presence of the three synonymous nucleotide changes.

### In vitro anti-HIV-1 and SHIV assay

The HIV-1 and SHIV indicator MAGI-CCR-5 [29] cell line was transduced with lentiviral anti-HIV vectors at an MOI of 2.5. The cells were sorted by flow cytometry for EGFP expression to over 98% EGFP-expressing cells. For each vector, polyclonal cell lines were plated in triplicate and infected with approximately 1000 transducing units of either the dualtropic HIV-1 isolate (HIV89.6) or the X4 SHIV isolate (KU-1). The MAGI-CCR-5 cells were then evaluated for SHIV and HIV-1 replication 48 hours after infection as described [28]. The fold-reduction in infected cells was calculated as the number of infected beta-galactosidase-expressing foci relative to the number of infected beta-galactosidase-expressing foci for the control vector RSC-SMPGW, for each anti-HIV vector.

### Analysis of transgene expression in vitro and in vivo

The above sorted MAGI-CCR-5 cells were analyzed for C46 expression by trypsinizing 1×10^6^ cells and washing twice with Dulbecco's modified phosphate buffered saline (D-PBS), then blocking in D-PBS with 5% nonfat milk on ice for 10 minutes. The volume was brought to 100 µl, and 1 µg of 2F5 antibody was added (Polymun Scientific, Vienna, Austria) and incubated on ice for 30 min. The cells were washed twice with D-PBS, then incubated on ice for 30 min in 200 µl of a 1∶80 dilution of 0.5 mg/ml goat anti-human secondary antibody conjugated to R-phycoerythrin (Jackson ImmunoResearch, West Grove, PA) in D-PBS. The cells were washed twice with D-PBS and analyzed by flow cytometry. For detection of siRNA expression by Northern Blot, transduced MAGI-CCR-5 cells were sorted to over 98% EGFP-positive cells and total RNA was isolated using STAT-60 (Tel-Test, Friendswood, TX) reagent according to the manufacturer's recommendations. Ten µg of total RNA was run on a 10% TBE-Urea Ready Gel (Bio-Rad Laboratories, Hercules, CA) together with a size marker and then electro-transferred to a Hybond N^+^ membrane (GE Healthcare, Piscataway, NJ). The membrane was baked at 80°C for 2 hours and then prehybridized for 3 hours at 37°C in QuikHyb hybridization buffer (Stratagene, La Jolla, CA) with 100 µg/ml final concentration of salmon sperm DNA. The membrane was then hybridized overnight in QuikHyb hybridization buffer at 37°C with 5 pmol of oligonucleotide DNA probe end labeled with γ-[^32^P]-ATP using 10 units of polynucleotide kinase (Invitrogen, Carlsbad, CA). The *tat/rev* site I shRNA probe used was 5′-GCGGAGACAGCGACGAAGAG-3′. After hybridization the membrane was then briefly washed in 6x SSPE buffer (20X SSPE is 3 M NaCl, 0.2 M NaH2PO4, 0.02 M EDTA, pH 7.4), then exposed using a Molecular Dynamics Storage Phosphor Screen (GE Healthcare, Piscataway, NJ) for one week and analyzed using a Typhoon Trio Variable Mode Imager (GE Healthcare) and ImageQuant TL (GE Healthcare) software. To evaluate RNA loading the blots were stripped with 1% sodium dodecyl sulfate at 85°C for 30 min and washed, then hybridized as described above using a U6 snRNA probe 5′-TATGGAACGCTTCTCGAATT-3′.

For in vivo analysis of C46 mRNA expression total RNA was isolated from macaque PBMCs using STAT-60 (Tel-Test, Friendswood, TX, USA) reagent according to manufacturer's recommendations. cDNA was synthesized using SuperScript II Reverse Transcriptase (Invitrogen, Carlsbad, CA, USA) in a reaction containing 4 µl 5x First-Strand Buffer, 25 µg/ml Oligo(dT)_12–18_, 0.5 mM each dNTPs, 10 mM DTT, 40 units RNaseOUT (Invitrogen), 50 ng total RNA template and H_2_O to a final volume of 20 µl. cDNA was incubated with RNase H at 37°C for 20 min prior to real-time PCR. Real-time PCR assay was performed on a 7500 Real Time PCR System (Applied Biosystems, Foster City, CA, USA), using QuantiTect SYBR Green PCR kit (Qiagen, Valencia, CA, USA) in a reaction containing 2 µl template cDNA, 25 µl 2x QuantiTect SYBR Green PCR Mastermix, 300 nM of each forward and reverse C46 primers (Forward: 5′-CCAGATCTTGGATGGAGTGG–3′, Reverse: 5′-CTGCTCGTTCTTCTCCTGCT-3′) or β-Globin primers (Forward: 5′- CCTATCAGAAAGTGGTGGCTGG-3′, Reverse: 5′-TTGGACAGCAAGAAAGTGAGCTT-3′) and H_2_O to a final volume of 50 µl. Reaction conditions were 50°C for 2 min and 95°C for 15 min (1×), then 94°C for 15 sec, 56°C for 30 sec, 72°C for 1 min (40×). A final dissociation curve analysis was performed to detect nonspecific amplification products.

### Pigtailed macaque transplantation

Healthy juvenile pigtailed macaques (*Macaca nemestrina*) were housed at the University of Washington National Primate Research Center under conditions approved by the American Association for Accreditation of Laboratory Animal Care. Study protocols were approved by the FHCRC Institutional Review Board and the FHCRC Institutional Animal Care and Use Committee. The conditions for transplantation have been described previously [14]. Briefly, animals were administered rhG-CSF at 100 µg/kg, and also given recombinant human stem cell factor at 50 µg/kg prior to bone marrow harvest. CD34^+^ cells were enriched by magnetic beads (Miltenyi Biotec, Auburn, CA) according to the manufacturer's instructions. All animals received myeloablative total-body irradiation and received rhG-CSF and standard supportive care after transplantation.

### MGMT-mediated selection of transduced macaque repopulating cells and in vivo tracking of marked cells

Monkeys received an intravenous infusion of 120 mg/m^2^ O6BG and 45 minutes to 1 hour later received an intravenous infusion of BCNU between 17.5 to 25 mg/m^2^. An identical dose of O6BG was given intravenously 8 hours after the end of BCNU infusion [31]. To track marking in vivo, leukocytes were analyzed as previously described [14]. Briefly, leukocytes isolated by ammonium chloride red cell lysis from heparinized peripheral blood and bone marrow samples drawn at multiple time points after transplantation were analyzed for EGFP/EYFP expression on a FACSVantage or FACSCanto (Becton–Dickinson, San Jose, CA, USA). The antibodies used for lineage-specific markers included CD3 (clone SP34-2), CD4 (clone L200), CD8 (clone RPA-T8), CD14 (clone M5E2), CD13 (clone L138), CD20 (clone L27), and CD34 (clone 563). All antibodies were supplied by Becton Dickinson (Franklin Lakes, NJ) and conjugated to phycoerythrin. Quantitative PCR analysis of marking was performed using primers and probes specific for EGFP or EYFP as previously described [32].

### Ex vivo anti-SHIV assay in macaque hematopoietic repopulating cell-derived CD4^+^ cells

CD4^+^ cells were enriched from 20 ml of macaque peripheral blood from a control animal T04228, which was transplanted with cells transduced with the control vector RSC-SMPGW and also from animal M05189, which received cells transduced with the RSC-SC46-IMPGW vector that expresses the C46 fusion inhibitor. Peripheral blood CD4^+^ cells were isolated using the Dynal CD4 Positive Isolation Kit (Invitrogen, Carlsbad CA) following the manufacturer's instructions. The recovered cells were cultured in Iscove's Modified Dulbecco's Medium (IMDM) supplemented with 10% fetal bovine serum (FBS) and were activated by the addition of paramagnetic beads (Dynabeads M-450 Tosylactivated, Invitrogen, Carlsbad CA) coated with mouse monoclonal antibodies anti-human CD3 (clone SP34-2 BD Biosciences, San Jose CA) and anti-human CD28 (CD28.2 obtained from Dr. Daniel Olive, INSERM, France, through the NIH Nonhuman Primate Reagent Resource). The beads were prepared according to the manufacturer's protocol and 1×10^7^ beads were coated with 0.5 µg of anti-CD3 and 4.5 µg of anti-CD28. The CD4^+^ purified cells were stimulated with 3 beads per cell and 100 U/ml recombinant human IL-2 (rhIL-2) (Chiron, Emeryville CA). One day after expansion the cells were plated at 2×10^4^ cells in a 48-well microplate and mock infected or challenged with SHIV in triplicate. Replication of SHIV was confirmed by MAGI-CCR-5 assay as described above. Twelve days after challenge, the percentage of EGFP expressing cells (EGFP) in infected and control cultures was determined by flow cytometry and the relative vector proviral copy number was determined by real-time PCR using primers specific for the lentiviral *cis*-acting region as described previously [14].

## Results

### Lentiviral anti-HIV MGMT vectors

**Figure 1:**
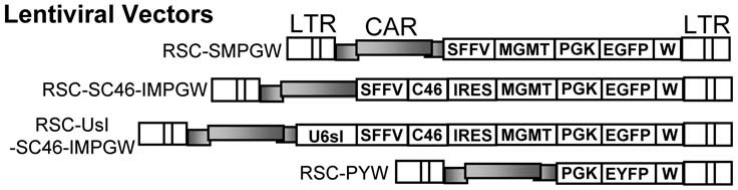
Lentiviral anti-HIV vectors. The lentiviral vector RSC-SMPGW contains an SFFV promoter (S) driving expression of the P140K mutant of MGMT (M) and also contains a human PGK promoter (P) driving expression of EGFP (G). This vector also contains a safety modified woodchuck hepatitis virus post transcriptional regulatory element (W). The following anti-HIV transgenes were added to this vector; the transmembrane-localized C46 HIV envelope fusion inhibitor expressed from the SFFV promoter with an IRES expressing MGMT (RSC-SC46-IMPGW), and the U6 driven *tat/rev* site I shRNA combined with the C46-expressing vector (RSC-UsI-SC46-IMPGW). The long terminal repeats (LTR) and lentiviral *cis*-acting region (CAR) are also indicated.

The control bicistronic lentiviral vector RSC-SMPGW has been described previously and contains the P140K version of MGMT for in vivo selection driven from an SFFV promoter, and an EGFP reporter gene driven from the human PGK promoter [14]. This vector also contains a safety-modified woodchuck hepatitis virus post-transcriptional regulatory element to enhance gene expression in vivo (**Figure 1**). We chose this configuration because in previous experiments we have observed efficient MGMT-based selection at the stem cell level using the SFFV promoter [19], [33], and we have also observed efficient EGFP expression using the PGK promoter in both myeloid and lymphoid lineages [14], [34]. The RSC-SC46-IMPGW vector expresses the C46 membrane-bound HIV-1 fusion inhibitor. For this C46 transgene, the signal peptide of the low-affinity nerve growth factor receptor (LNGFR) directs translocation into the endoplasmic reticulum, and the human immunoglobulin G2 (IgG2) hinge fused to the human CD34 membrane-spanning domain serves as a scaffold to anchor the peptide to the cell surface [23]. High level expression of the C46 fusion inhibitor is required for a potent antiviral effect [35], so we expressed C46 from the strong SFFV promoter and expressed MGMT from an IRES. We chose this configuration because expression from an IRES (MGMT) leads to lower levels of expression than the transgene expressed from promoter 5′ to the IRES [36]. Thus higher levels of C46 expression would be obtained relative to MGMT. Following selection with O6BG and BCNU, cells expressing lower levels of MGMT should have a selective disadvantage, and cells surviving BCNU selection might have even higher levels of C46 than unselected cells. The RSC-UsI-SC46-IMPGW vector has an additional Pol III-driven *tat*/*rev* specific site I shRNA [37]. The lentiviral vectors are based on the RRL vector system and are self-inactivating (SIN) and contain a central polypurine tract [38]. For these studies we chose two anti-HIV transgenes that inhibit both HIV-1 and SHIV, so anti-HIV vectors would be expected to have similar efficacy inhibiting SHIV in our macaque studies and inhibiting HIV-1 in potential future clinical applications. The C46 targets HIV-1 *env* and the site I shRNA targets HIV-1 *tat* and *rev* which are all in the SHIV chimera and required for SHIV replication.

### Lentiviral anti-HIV vectors potently inhibit SHIV and HIV-1 infection

**Figure 2:**
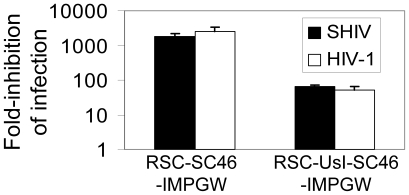
Inhibition of HIV-1 and SHIV in vitro. The anti-HIV vectors were evaluated for SHIV and HIV-1 inhibition using a single-cycle replication assay where MAGI-CCR-5 cells were transduced with the indicated vectors, sorted to over 98% EGFP-positive, then challenged with the indicated viruses and tested for virus replication 48 hours after challenge. The fold-reduction in detected infectious units is reported for each vector relative to the control vector RSC-SMPGW that does not contain an anti-HIV transgene.

**Figure 3:**
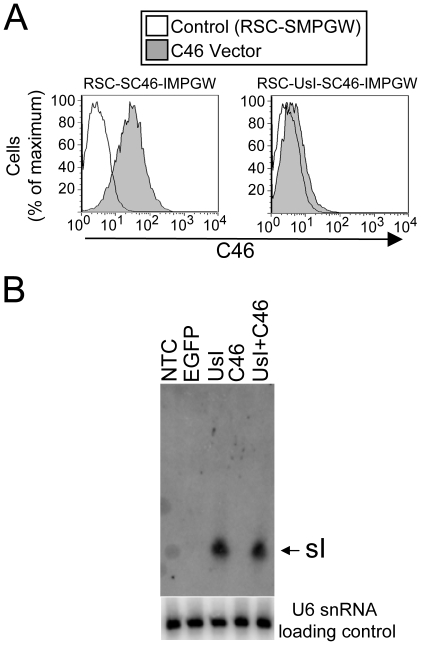
Effect of multiple components on expression of individual anti-HIV transgenes. (**A**) Expression of the membrane-bound C46 fusion inhibitor was detected by antibody staining in transduced MAGI-CCR-5 cells. Cells transduced with either a control vector RSC-SMPGW that does not express C46, or the C46 vector RSC-SC46-IMPGW, or the U6-driven site I shRNA and C46 vector RSC-UsI-SC46-IMPGW were sorted to over 98% EGFP-positive cells and stained using a primary C46 monoclonal and a secondary phycoerythrin-conjugated antibody. Expression of the C46 transgene is greatly reduced in cells transduced with the vector that also expresses the U6-driven site I *tat/rev* shRNA. (**B**) Expression of the U6-driven site I tat/rev shRNA was compared by Northern blot in MAGI-CCR-5 cells using a tat/rev site I-specific probe (top panel). NTC is non-transduced control, EGFP is cells transduced with a lentiviral vector that only expressed EGFP, UsI is cells transduced with a vector that expresses EGFP and the U6-driven site I shRNA, C46 is the vector RSC-SC46-IMPGW, UsI+C46 is the vector RSC-UsI-SC46-IMPGW. The arrow indicates approximately 21 nt. Expression of the U6-driven site I tat/rev shRNA was not affected by the addition of a C46 transgene. The bottom panel is a loading control with a U6 snRNA probe hybridized to the same blot.

We first compared the ability of the two anti-HIV vectors to inhibit SHIV and also HIV-1 using a single-cycle infection assay. In this assay the indicator MAGI-CCR-5 cell line was transduced with anti-HIV vectors and the cells were then sorted by flow cytometry for EGFP expression to over 98% percent EGFP-expressing cells. For each vector these polyclonal cell lines were challenged in triplicate with either SHIV or HIV-1 and infection was evaluated 48 hours after infection. The MAGI-CCR-5 cell line detects both SHIV and HIV infection, so this approach allowed us to quantitatively compare the relative ability of each vector to inhibit either HIV-1 or SHIV during only one round of infection. The fold-reduction in infected cells is reported relative to the control vector RSC-SMPGW for each vector (**Figure 2**). In this single-cycle infection test, the lentiviral vector expressing C46 provided approximately 1800-fold and 2500-fold inhibition of HIV-1 and SHIV replication, respectively. The lentiviral vector that also contained the U6-driven tat/rev shRNA inhibited both HIV-1 and SHIV replication to a lesser extent, 66-fold and 50-fold, respectively. For both vectors the difference between inhibition of HIV-1 and inhibition of SHIV was not significant (p = 0.49, p = 0.35) as would be expected since these vectors target HIV-1 *tat*, *rev* and *env* that are in both HIV-1 and also in SHIV. We also evaluated the effect of inclusion of multiple anti-HIV transgenes on the individual genes (**Figure 3**). We found that adding the U6-driven site I tat/rev shRNA decreased C46 expression, consistent with our findings that the combinatorial vector RSC-UsI-SC46-IMPGW was not as effective as RSC-SC46-IMPGW in the SHIV and HIV-1 inhibition assay. Conversely, the C46 transgene did not affect expression of the U6-driven site I shRNA as assessed by Northern blot.

### Development of a lentiviral helper plasmid with a modified *rev* for production of lentiviral vectors which express the site I *tat/rev* shRNA

**Figure 4:**
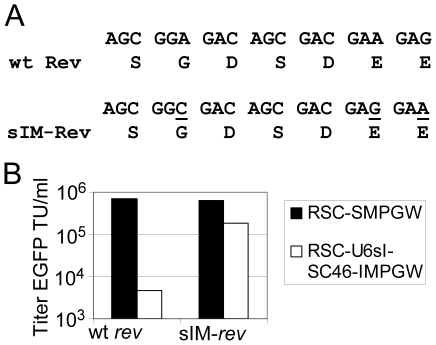
Production of *tat/rev* shRNA-containing lentiviral vectors at high titer using a modified helper plasmid. (**A**) Three synonymous mutations indicated by an underline were introduced into *rev* in the lentiviral helper plasmid pCMVRdelta8.74 which expresses Gag-Pol and Rev to create pCMVRdelta8.74-sIM, which contains three mismatches at the *tat/rev* site I shRNA target sequence. (**B**) Lentiviral vectors were prepared by transient transfection using either the lentiviral helper plasmid pCMVRdelta8.74, which contains the wild-type *rev* sequence (wt-*rev*), or the pCMVRdelta8.74-sIM helper, which contains the mutated (sIM-*rev*) and titered using the EGFP reporter. The RSC-UsI-SC46-IMPGW vector containing the tat/rev shRNA is produced at low titer using the wt *rev* helper, but is produced at 38-fold higher titer using the sIM-*rev* helper.

Lentiviral vectors can be produced at high titer and mediate efficient transduction of human hematopoietic repopulating cells in murine xenotransplantation assays [39] and also canine [25] and pigtailed macaque repopulating cells [14]. However, one challenge of using lentiviral vectors for AIDS gene therapy is that anti-HIV transgenes that interfere with HIV-1 replication steps required for efficient HIV-based lentiviral vector production can severely reduce titers [40], [41]. We were able to produce the anti-HIV lentiviral vector containing the C46 transgene RSC-SC46-IMPGW at high titer; however, the titers were drastically reduced for the RSC-UsI-SC46-IMPGW vector, which contains the *tat/rev* shRNA expression cassette. The lentiviral vectors we used are pseudotyped with VSV-G, so the C46 transgene which inhibits fusion between the HIV-1 envelope glycoprotein and the cell membrane would not be expected to reduce lentiviral vector titers, and did not. However, the site I *tat/rev* shRNA, targets *rev,* and Rev protein is required for efficient production of lentiviral vector virions, likely explaining the low titers of the site I *tat/rev* shRNA. The titer of foamy vector virions that contain this *tat/rev* shRNA is not adversely affected [42], so we reasoned that knockdown of lentiviral vector RNAs by cleavage of the shRNA target in the lentiviral vector was likely not the main mechanism for the low titers observed since this mechanism would also be expected to reduce foamy vector titers. Instead, we considered that the main mechanism may be knockdown of Rev function [43] or of Gag-Pol helper function via shRNA-mediated degradation of helper plasmid transcripts that include the *rev* target sequence. The lentiviral helper plasmid pCMVRdelta8.74 produces Gag, Pol, and Rev which are all required for production of lentiviral vector virions. The *tat/rev* shRNA target sequence is located on both *gag-pol* and on *rev* mRNAs so we reasoned that inserting a synonymous mutation in *rev* that would alter the shRNA target on the lentiviral helper plasmid, but would not change the Rev ORF, might increase titers. A 3 bp synonymous mutation was incorporated in *rev* at the site I in pCMVRdelta8.74 helper plasmid (**Figure 4A**), and the resulting helper plasmid was named pCMVRdelta8.74-sIM. When we produced the *tat/rev* shRNA containing vector, RSC-UsI-SC46-IMPGW, using this helper plasmid with the mutated site I *rev*, the vector titers increased 38-fold (**Figure 4B**). The titer of the control vector RSC-SMPGW was slightly decreased (13%), suggesting the *rev* mutation minimally impaired the efficiency of lentiviral vector production. Using the pCMVRdelta8.74-sIM helper plasmid, we were able to produce the *tat/rev* shRNA-containing lentiviral vector RSC-UsI-SC46-IMPGW at unconcentrated titers in excess of 10^5^ and at titers in excess of 10^7^ transducing units/ml after 100-fold concentration, titers sufficient for experiments in large animal models and also for clinical studies.

### Transduction and engraftment of lentiviral anti-HIV vectors in the pigtailed macaque

**Figure 5:**
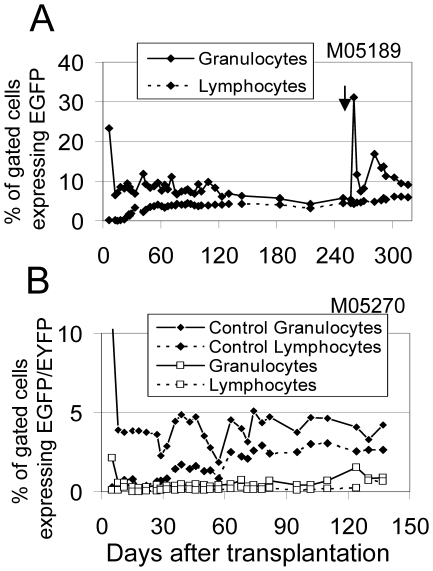
Transgene expression levels in peripheral blood cells of macaques and MGMT-mediated in vivo selection using O6BG and BCNU. The percentages of EGFP and EYFP-expressing leukocytes detected by flow-cytometry are shown for monkeys M05189 (**A**) and M05270 (**B**). Granulocytes and lymphocytes were gated based on forward and side-scatter properties and the percentage of EGFP or EYFP-expressing gated cells is shown. Administration of O6BG and BCNU to macaque M05189 is indicated by an arrow. For animal M05270 the control arm vector transduced cells are shown with back diamonds connected by a solid line (granulocytes) or dotted line (lymphocytes), and anti-HIV vector RSC-UsI-SC46-IMPGW transduced cells are shown by open boxes connected by a solid line (granulocytes) or dotted line (lymphocytes).

In order to translate an AIDS gene therapy approach to the clinic, it is important to determine if the proposed anti-HIV transgenes might have a deleterious effect on hematopoietic stem cell engraftment and differentiation. We have previously shown that the pigtailed macaque (*M. nemestrina*) is an excellent model to evaluate lentiviral HSC gene therapy strategies because high level marking in multiple hematopoietic lineages can be obtained [14], likely due to the absence of TRIM5α restriction [15]. We have previously demonstrated efficient marking into myeloid and lymphoid lineages using the control vector RSC-SMPGW [14], so we performed two transplants with the anti-HIV vectors. The pre-transplant marking and engraftment data for these two animals is shown in **Table 1**. The first transplant was with the RSC-SC46-IMPGW vector that expresses the C46 membrane-bound HIV-1 fusion inhibitor. In this animal (M05189) once marking had stabilized, approximately 5% and 4% of granulocytes and lymphocytes were gene-modified as determined by EGFP expression (**Figure 5**). Marking was slightly lower than what we had achieved previously in two other macaques using the RSC-SMPGW control vector, which does not have an anti-HIV cassette [14] but was still significant and stable.

**Table 1:** Lentiviral transduction and engraftment of pigtailed macaque CD34^+^ cells.

Monkey*	CD34+ Purity	No. of CD34-enriched cells/kg ×10^6^ before culture	Transgenes	No. of infused cells/kg ×10^6^	MOI†	Pre-infusion transduction efficiency‡	Days to ANC >500	Days to platelets >20,000
M05189 (4.8 kg)	95%	48.5	C46, MGMT	22.7	5	49.7%	17	34
M05270 (4.8 kg)	94%	30	EYFP	34.2	2	20.6%	>150	18
		30	EGFP, MGMT, U6shI, C46	40.4	2	6.28%		

* Monkey and weight at time of transplantation in brackets.

† Multiplicity of infection based on titer determined by transduction of HT1080 cells.

‡ Percentage of fluorescence-positive cells assessed by flow cytometry for EGFP or EYFP in liquid cultures on day 11 (M05270) or 6 (M05189) after transduction.

In the second transplant we employed a competitive repopulation approach where the anti-HIV vector RSC-UsI-SC46-IMPGW that expresses EGFP was evaluated with a second experimental arm using the control vector RSC-PYW that expresses only EYFP. Macaque CD34^+^ cells were separated into two experimental arms with equal numbers of CD34^+^ cells that were exposed to each vector separately. Following the ex vivo exposure to the vector preparations, the cells were washed extensively and then re-infused sequentially so that macaque repopulating cells can be marked with either vector, but not with both. This approach allowed us to evaluate the relative marking between the control EYFP and the anti-HIV EGFP vector in the same macaque, thereby eliminating inter-animal variability. It is important to note that, since each experimental arm represents only one-half of the infused cells, the marking observed for each individual arm is only one-half of what would be expected if the transplant were performed using only a single vector. For example, the marking in granulocytes in the control EYFP arm for M05270, which was approximately 4.5% after marking stabilized (30 to 120 days), would be expected to be approximately 9% if this were the only vector used. Thus, the marking level in the control EYFP experimental arm of M05270 closely matched what we observed in macaque M05189, which received the vector that expresses the C46 transgene and MGMT in addition to the EGFP transgene. For the EGFP-expressing anti-HIV vector arm of macaque M05270, we observed much lower marking in granulocytes, approximately 0.6%. The marking in lymphocytes gradually rose to a plateau for both M05189 and for the control EYFP arm in animal M05270. This slow rise in lymphocyte marking was similar to what we have previously observed for animals with no anti-HIV transgene cassette [14].

The pre-transplant marking (**Table 1**) for macaque M05270 showed there was an approximately 3.3-fold lower marking rate for the EGFP-positive anti-HIV vector relative to the control EYFP-positive vector, despite being matched for MOI based on transduction in HT1080 cells. In vivo, marking with the anti-HIV vector was approximately 7.5-fold lower than the control EYFP arm. Analysis of the marking in vivo in M05270 by quantitative PCR confirmed the lower marking in the EGFP anti-HIV arm, with 11.4-fold lower marking relative to the control arm, showing that the difference was not due to silencing of the anti-HIV vector. The approximately two-fold loss in gene-marking from pre-transplant levels to marking in repopulating cells and the overall 7.5 fold lower transduction rate in macaque repopulating cells relative to transduction of the HT1080 cells used to determine the titer of vector preparations suggests there may be a deleterious effect of the U6-driven site I *tat*/*rev* shRNA in primate repopulating cells. For the RSC-UsI-SC46-IMPGW vector, marking in lymphocytes remained low until day 111 after transplantation and then increased slightly to match marking in granulocytes.

**Figure 6:**
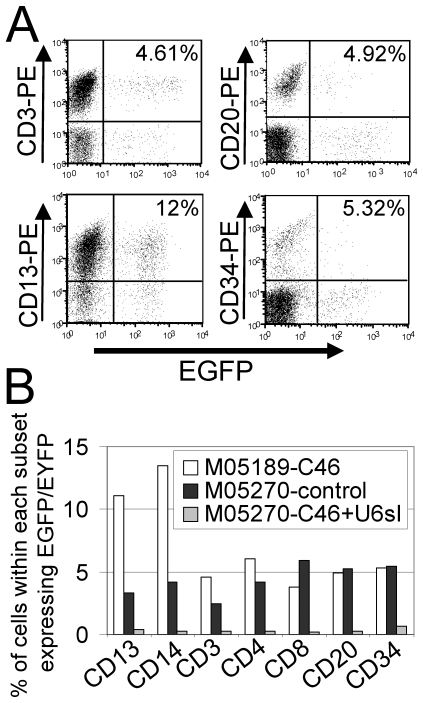
Flow-cytometric analysis of transgene-expressing cells in peripheral blood subpopulations and bone marrow CD34^+^ cells. (**A**) Displayed is the percentage of transgene positive cells in different leukocyte subpopulations in the peripheral blood of monkey M05189 that received the C46 membrane fusion inhibitor and EGFP-expressing vector. In this macaque EGFP-expressing cells were found at significant percentages in all lineages examined. (**B**) Lineage-specific staining for macaques M05189 and M05270. Macaque M05189 and the control EYFP arm of M05270 had significant percentages of transgene-expressing cells in all lineages. For the experimental EGFP-expressing arm of M05270 that received the vector with the C46 fusion inhibitor and the U6-driven site I shRNA, the percentage of transgene-expressing cells was low in all lineages examined.

The relative marking levels in myeloid (CD13, CD14) or lymphoid (CD3, CD4, CD8, and CD20) subsets and also in bone marrow CD34^+^ cells was also evaluated (**Figure 6**). The EGFP marker allowed us to accurately assess marking in myeloid and lymphoid lineages. For macaque M05189 that received the membrane-bound C46 HIV-1 fusion inhibitor, relatively high marking levels were detected in all lineages examined. There was no obvious difference in the relative marking in these lineages compared to historical control macaques [14] that received the RSC-SMPGW vector, providing strong evidence that the C46 transgene does not have any obvious lineage-specific effects on differentiation. We confirmed expression of the C46 in vivo in peripheral blood from monkey M05189 by real-time reverse transcriptase PCR. The data showed 12.6-fold higher expression of C46 mRNA in sorted EGFP-positive cells from M05189 relative to sorted EGFP-negative cells. Analysis of C46 mRNA expression in EGFP positive or negative cells from the control monkey T04228 that received the control vector RSC-SMPGW showed only background levels. For macaque M05270 the marking level for the control EYFP arm was relatively high in all lineages examined, but marking for the EGFP-expressing anti-HIV vector was much lower in all lineages examined.

### MGMT-mediated increase in marking of a lentiviral anti-HIV vector

The MGMT cassette in the anti-HIV vectors provides a means to increase marking after transplantation. The P140K mutant MGMT is not sensitive to the inhibitor O6BG, which is used to inactivate endogenous MGMT. The alkylating agent BCNU is toxic to cells that do not express MGMT, so in the presence of both agents cells that do not express the mutant P140K MGMT are killed resulting in an increase in the percentage of marked EGFP-expressing cells. We treated macaque M05189 with O6BG and BCNU in an attempt to increase marking with the anti-HIV vector that expresses the C46 fusion inhibitor. We observed a large but transient increase in marking in granulocytes that began to stabilize at approximately 9% up from a pre-transplant marking at approximately 4–5% (**Figure 5**). Following the treatment the marking in lymphocytes slowly increased from approximately 4% to approximately 7%.

### Protection of macaque lymphocytes from SHIV infection after HSC transplantation

**Figure 7:**
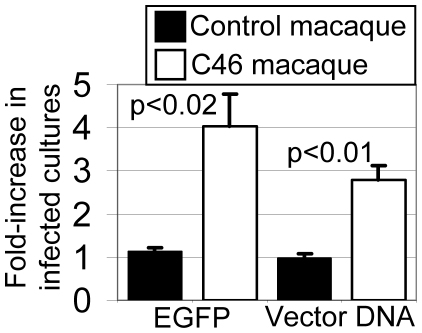
Macaque repopulating CD4^+^ lymphocytes that express the C46 fusion inhibitor are protected from SHIV infection as evidenced by enrichment of transduced cells during ex vivo challenge. CD4-enriched peripheral blood lymphocytes from macaque T04228 transplanted with CD34^+^ cells transduced with the control EGFP expressing vector RSC-SMPGW (Control macaque) or macaque M05189 transduced with the lentiviral C46 and EGFP expressing vector RSC-SC46-IMPGW (C46 Macaque) were either mock infected or challenged with SHIV in triplicate. Twelve days after challenge the percentage of EGFP expressing cells (EGFP) in infected and control cultures was determined by flow cytometry. The relative vector proviral copy number (Vector DNA) was also determined by real-time PCR to assess the enrichment of transduced cells in infected cultures. Standard error and p values from a Student's t test are shown.

The goal of AIDS gene therapy is to provide patients with mature HIV-resistant immune cells derived from repopulating HSCs. To be effective, mature immune cells differentiated in vivo from long-term hematopoietic repopulating cells must retain their resistance to viral infection. To evaluate the viral resistance of immune cells differentiated in vivo, we isolated CD4^+^ lymphocytes from macaque M05189 peripheral blood and challenged these cells with SHIV ex vivo. We isolated and challenged CD4^+^ cells after MGMT-mediated selection when marking in lymphocytes in M05189 had risen to 7%, reasoning that this should provide a sufficient percentage of EGFP-positive cells to observe an enrichment if cells were protected during an ex vivo challenge. As a control we isolated peripheral blood cells from a previously described control macaque T04228 [14] that was transplanted with cells transduced with the RSC-SMPGW vector that does not contain an anti-HIV transgene. T04228 had also been previously treated with O6BG and BCNU to increase marking. Macaque lymphocytes were stimulated with beads coated with CD3 and CD28 antibody and challenged at an MOI of 0.05 in triplicate. Twelve days later the percentage of EGFP cells was determined and the fold-increase in the percentage of cells expressing EGFP was determined relative to a mock-infected culture to determine if EGFP-expressing cells with the anti-HIV vector cells had been protected and therefore enriched (**Figure 7**). We observed a statistically significant (p<0.02) 4-fold increase in EGFP-expressing cells in challenged cells from macaque M05189 that also expressed the C46 HIV fusion inhibitor but not in cells from the control macaque T04228. The percentage of EGFP-expressing cells increased from 8.3% to 33%. DNA was isolated from these cultures and real-time PCR was performed using a lentiviral vector-specific primer and probe set to verify the enrichment of gene-modified cells. Analysis of the relative proviral vector content between mock-challenged and challenged cells confirmed that there was a statistically significant (p<0.01) enrichment of gene-modified cells in the challenged cultures for M05189 but not for the control macaque T04228. Thus we have shown that mature lymphocytes derived from long-term repopulating cells can be selected using MGMT and are protected from challenge.

## Discussion

Here we developed lentiviral anti-HIV vectors that also contain a P140K MGMT transgene cassette and evaluated these vectors in in vitro studies and also in a clinically relevant primate model for AIDS gene therapy. We found that vectors containing a C46 HIV fusion inhibitor did not adversely affect the lentivirus titer, but that the addition of an anti-HIV siRNA transgene led to significant reduction in titer. To overcome the dramatic reduction in the titer of anti-HIV siRNA lentivirus vectors, we developed a modified *rev* helper plasmid that allows for high titer lentivirus preparations containing anti-HIV siRNA transgenes. Using fluorescent markers we were able to accurately track marking in vivo. We observed efficient gene transfer using a lentiviral vector that expresses a membrane-anchored HIV-1 fusion inhibitor and demonstrated that lymphocytes that are differentiated in vivo from engrafted hematopoietic repopulating cells transduced with this anti-HIV vector were protected from SHIV infection.

HSC gene therapy has the potential to inhibit the progression of, or potentially cure AIDS. However, obtaining efficient gene delivery to HSCs has been a roadblock for this field, so developing means for efficient gene transfer of anti-HIV transgenes to HSCs is an important step towards the goal of an effective AIDS gene therapy. There are several models for HIV gene therapy including mouse xenotransplantation models [44], [45]. However, viral replication and AIDS are best modeled in nonhuman primates where several of the pathological consequences of AIDS can be reproduced. Simian AIDS pathology includes high viral load in the peripheral circulation, hematopoietic abnormalities and loss of CD4^+^ lymphocytes in the peripheral blood, and in the gut-associated lymphoid tissue. In the SHIV macaque model the similarity of the pathology of simian AIDS to human AIDS extends to encephalitis and other organ-specific disease [16]. Because of these advantages the nonhuman primate AIDS model has played a crucial role in the development of anti-HIV treatment strategies [46]-[48]. In the SHIV macaque model, unlike the human population, the timing of infection can be controlled, and findings can be quantified and directly correlated with disease under controlled conditions. For these reasons the SHIV-macaque model is also an excellent model to study AIDS gene therapy strategies. The competitive repopulation assay we describe here using EGFP and EYFP fluorescent markers has allowed us to compare marking across multiple hematopoietic lineages.

Lentiviral vectors have several advantages for HSC gene therapy including the fact that they transduce quiescent cells more efficiently than gammaretroviral vectors and they can be produced at high titer in a self-inactivating (SIN) configuration [49]. Lentiviral vectors may also be safer than gammaretroviral vectors [50] based in part on differences in their integration profile [51], [52]. One disadvantage of using HIV-1 based lentiviral vectors for AIDS gene therapy is that some anti-HIV transgenes that target components of HIV involved in vector production can reduce vector titers [40], [41]. This is generally not a problem for in vitro studies or mouse studies where a limited number of target cells need to be transduced. However, for preclinical monkey studies or for clinical trials, obtaining high titers is critical to establishing high marking levels. Here we show that mutating the lentiviral helper plasmid by making a synonymous mutation in *rev* allowed for production of a high titer combinatorial HIV-based lentiviral anti-HIV vector containing a potent anti *tat/rev* shRNA. Importantly, this allowed us to generate large-scale vector preparations with this anti *tat*/*rev* vector at a titer sufficient to perform primate transplantation experiments.

Stable resistance to SHIV replication is a prerequisite for an effective gene therapy, but it is also critical that the transgene does not impair engraftment or differentiation of transplanted HSCs. We obtained high levels of engraftment in a monkey model with the C46 membrane-bound fusion inhibitor. For this vector there were no obvious differences in EGFP expression between myeloid or lymphoid lineages or within subsets of cell types within these lineages, suggesting that the C46 fusion inhibitor should also be safe for use in a clinical setting. Conversely, we observed low marking when the anti-HIV vector also contained a shRNA driven by a U6 Pol III promoter. Our in vivo data are consistent with data reported by An et al. [53] where expression of shRNAs from the U6 Pol III promoter inhibited the expansion of primate lymphocytes in vitro.

The anti-HIV vectors that we developed here also contain the P140K mutant MGMT transgene to allow for in vivo selection. We have previously shown that MGMT supports efficient in vivo selection of HSCs in large animal models [18], [19]. Here we show in the macaque that gene marking of an anti-HIV vector can be increased in both myeloid and lymphoid populations. Although we have only shown protection in one macaque we have established that stable MGMT-mediated selection is reproducible in other monkeys (H-P. Kiem and G.D. Trobridge, unpublished data). Thus we believe this approach to increase the percentage of protected cells in vivo will likely be reproducible, and this model will be a powerful way to test anti-HIV vectors prior to clinical trials. Treatment-associated abnormalities associated with O6BG and BCNU were minimal with transient transaminitis that resolved quickly following treatment. This suggests that MGMT-mediated in vivo selection of anti-HIV gene-modified cells should be well tolerated, and HIV patients who suffer from HIV-associated lymphoma in particular could benefit from this approach. Importantly, we show that following engraftment, MGMT selection, and differentiation in vivo, these cells were protected from SHIV infection. The P140K MGMT is thus a very useful tool in this model that has allowed us to increase the marking in vivo. Using this strategy, marking could be further increased with additional O6BG and BCNU treatments, and monkeys could be challenged with SHIV. These studies should be highly informative to establish a proof-of-concept for AIDS gene therapy, and will facilitate evaluation and improvement of vectors being considered for clinical applications. Using the competitive repopulation model we describe here, we plan to test additional combinatorial vectors we have recently constructed that contain H1 Pol III and also Pol II promoters for shRNA expression which may be safer in terms of off-target effects on endogenous microRNAs [53]-[55]. An important feature of the SHIV pigtailed macaque model is that anti-HIV-1 transgenes can be assessed, so efficacy in this model should translate well to clinical studies. Additionally, because of the defective TRIM5α in the pigtailed macaque, we were able to use HIV-derived vectors that efficiently transduce human CD34^+^ cells, unlike SIV-derived vectors [56]. Thus, after removing the PGK-EGFP cassette from a vector with demonstrated efficacy, we plan to evaluate the vector in a clinical setting of AIDS lymphoma. After transplantation with transduced cells, treatment with O6BG and BCNU would not only have an anti-leukemic effect but should also increase the number of cells protected from HIV-1.

## References

[1] (2008) 07 AIDS Epidemic Update..

[2] Yeni PG, Hammer SM, Carpenter CC, Cooper DA, Fischl MA (2002). Antiretroviral treatment for adult HIV infection in 2002: updated recommendations of the International AIDS Society-USA Panel (Review) [erratum appears in JAMA. 2003 Jan-Feb;11(1):32].. JAMA.

[3] Carr A (2003). Toxicity of antiretroviral therapy and implications for drug development (Review).. Nature Reviews Drug Discovery.

[4] Klausner RD, Fauci AS, Corey L, Nabel GJ, Gayle H (2003). Medicine. The need for a global HIV vaccine enterprise.. Science.

[5] (2007). Cold shower for AIDS vaccines (Editorial).. Nat Med.

[6] Buchschacher GL, Wong-Staal F (2001). Approaches to gene therapy for human immunodeficiency virus infection (Review).. Hum Gene Ther.

[7] Strayer DS, Branco F, Landre J, BouHamdan M, Shaheen F (2002). Combination genetic therapy to inhibit HIV-1.. Molecular Therapy.

[8] Rossi JJ, June CH, Kohn DB (2007). Genetic therapies against HIV (Review).. Nat Biotechnol.

[9] Dropulic B, June CH (2006). Gene-based immunotherapy for human immunodeficiency virus infection and acquired immunodeficiency syndrome (Review).. Hum Gene Ther.

[10] Hutter G, Nowak D, Mossner M, Ganepola S, Mussig A (2009). Long-term control of HIV by CCR5 Delta32/Delta32 stem-cell transplantation.. N Engl J Med.

[11] Bauer G, Selander D, Engel B, Carbonaro D, Csik S (2000). Gene therapy for pediatric AIDS (Review).. Ann NY Acad Sci.

[12] Amado RG, Mitsuyasu RT, Rosenblatt JD, Ngok FK, Bakker A (2004). Anti-human immunodeficiency virus hematopoietic progenitor cell-delivered ribozyme in a phase I study: myeloid and lymphoid reconstitution in human immunodeficiency virus type-1-infected patients.. Hum Gene Ther.

[13] Mitsuyasu RT, Merigan TC, Carr A, Zack JA, Winters MA (2009). Phase 2 gene therapy trial of an anti-HIV ribozyme in autologous CD34+ cells.. Nat Med.

[14] Trobridge GD, Beard BC, Gooch C, Wohlfahrt M, Olsen P (2008). Efficient transduction of pigtailed macaque hemtopoietic repopulating cells with HIV-based lentiviral vectors.. Blood.

[15] Brennan G, Kozyrev Y, Kodama T, Hu S-L (2007). Novel TRIM5 isoforms expressed by *Macaca nemestrina*.. J Virol.

[16] Joag SV (2000). Primate models of AIDS (Review).. Microbes & Infection.

[17] Davis BM, Roth JC, Liu L, Xu-Welliver M, Pegg AE (1999). Characterization of the P140K, PVP(138–140)MLK, and G156A O6-methylguanine-DNA methyltransferase mutants: implications for drug resistance gene therapy.. Hum Gene Ther.

[18] Neff T, Beard BC, Peterson LJ, Anandakumar P, Thompson J (2005). Polyclonal chemoprotection against temozolomide in a large-animal model of drug resistance gene therapy.. Blood.

[19] Neff T, Horn PA, Peterson LJ, Thomasson BM, Thompson J (2003). Methylguanine methyltransferase-mediated in vivo selection and chemoprotection of allogeneic stem cells in a large-animal model.. J Clin Invest.

[20] Trobridge G, Beard BC, Kiem H-P (2005). Hematopoietic stem cell transduction and amplification in large animal models.. Hum Gene Ther.

[21] Crone TM, Goodtzova K, Edara S, Pegg AE (1994). Mutations in human O6-alkylguanine-DNA alkyltransferase imparting resistance to O6-benzylguanine.. Cancer Res.

[22] Xu-Welliver M, Kanugula S, Pegg AE (1998). Isolation of human O6-alkylguanine-DNA alkyltransferase mutants highly resistant to inactivation by O6-benzylguanine.. Cancer Res.

[23] Egelhofer M, Brandenburg G, Martinius H, Schult-Dietrich P, Melikyan G (2004). Inhibition of human immunodeficiency virus type 1 entry in cells expressing gp41-derived peptides.. J Virol.

[24] Li MJ, Bauer G, Michienzi A, Yee JK, Lee NS (2003). Inhibition of HIV-1 infection by lentiviral vectors expressing Pol III-promoted anti-HIV RNAs.. Molecular Therapy.

[25] Horn PA, Keyser KA, Peterson LJ, Neff T, Thomasson BM (2004). Efficient lentiviral gene transfer to canine repopulating cells using an overnight transduction protocol.. Blood.

[26] Salter RD, Howell DN, Cresswell P (1985). Genes regulating HLA class I antigen expression in T-B lymphoblast hybrids.. Immunogenetics.

[27] Howell DN, Andreotti PE, Dawson JR, Cresswell P (1985). Natural killing target antigens as inducers of interferon: studies with an immunoselected, natural killing-resistant human T lymphoblastoid cell line.. J Immunol.

[28] Kimpton J, Emerman M (1992). Detection of replication-competent and pseudotyped human immunodeficiency virus with a sensitive cell line on the basis of activation of an integrated beta-galactosidase gene.. J Virol.

[29] Chackerian B, Long EM, Luciw PA, Overbaugh J (1997). Human immunodeficiency virus type 1 coreceptors participate in postentry stages in the virus replication cycle and function in simian immunodeficiency virus infection.. J Virol.

[30] Collman R, Balliet JW, Gregory SA, Friedman H, Kolson DL (1992). An infectious molecular clone of an unusual macrophage-tropic and highly cytopathic strain of human immunodeficiency virus type 1.. J Virol.

[31] Kreklau EL, Liu N, Li Z, Cornetta K, Erickson LC (2001). Comparison of single- versus double-bolus treatments of O(6)-benzylguanine for depletion of O(6)-methylguanine DNA methyltransferase (MGMT) activity in vivo: development of a novel fluorometric oligonucleotide assay for measurement of MGMT activity.. J Pharmacol Exp Ther.

[32] Jung CW, Beard BC, Morris JC, Neff T, Beebe K (2007). Hematopoietic stem cell engraftment: a direct comparison between intramarrow and intravenous injection in nonhuman primates.. Exp Hematol.

[33] Gerull S, Beard BC, Peterson LJ, Neff T, Kiem H-P (2007). *In vivo* selection and chemoprotection after drug resistance gene therapy in a nonmyeloablative allogeneic transplantation setting in dogs.. Hum Gene Ther.

[34] Kiem H-P, Allen J, Trobridge G, Olson E, Keyser K (2007). Foamy virus-mediated gene transfer to canine repopulating cells.. Blood.

[35] Hermann FG, Martinius H, Egelhofer M, Giroglou T, Tonn T (2009). Protein scaffold and expression level determine antiviral activity of membrane-anchored antivital peptides.. Hum Gene Ther.

[36] Ngoi SM, Chien AC, Lee CG (2004). Exploiting internal ribosome entry sites in gene therapy vector design (Review).. Current Gene Therapy.

[37] Li M, Rossi JJ (2005). Lentiviral vector delivery of siRNA and shRNA encoding genes into cultured and primary hematopoietic cells.. Methods in Molecular Biology.

[38] Dull T, Zufferey R, Kelly M, Mandel RJ, Nguyen M (1998). A third-generation lentivirus vector with a conditional packaging system.. J Virol.

[39] Miyoshi H, Smith KA, Mosier DE, Verma IM, Torbett BE (1999). Transduction of human CD34+ cells that mediate long-term engraftment of NOD/SCID mice by HIV vectors.. Science.

[40] Mautino MR, Morgan RA (2000). Potent inhibition of human immunodeficiency virus type 1 replication by conditionally replicating human immunodeficiency virus-based lentiviral vectors expressing envelope antisense mRNA.. Hum Gene Ther.

[41] Bahner I, Sumiyoshi T, Kagoda M, Swartout R, Peterson D (2007). Lentiviral vector transduction of a dominant-negative Rev gene into human CD34+ hematopoietic progenitor cells potently inhibits human immunodeficiency virus-1 replication.. Molecular Therapy.

[42] Kiem H-P, Wu RA, Sun G, von Laer D, Rossi JJ (2009). Foamy combinatorial anti-HIV vectors with MGMTP140K potently inhibit HIV-1 and SHIV replication and mediate selection in vivo.. Gene Therapy.

[43] Brandt S, Blissenbach M, Grewe B, Konietzny R, Grunwald T (2007). Rev proteins of human and simian immunodeficiency virus enhance RNA encapsidation.. PLoS Pathogens.

[44] Berges BK, Wheat WH, Palmer BE, Connick E, Akkina R (2006). HIV-1 infection and CD4 T cell depletion in the humanized Rag2−/−gamma c−/− (RAG-hu) mouse model.. Retrovirology.

[45] Gorantla S, Sneller H, Walters L, Sharp JG, Pirruccello SJ (2007). Human immunodeficiency virus type 1 pathobiology studied in humanized BALB/c-Rag2−/−gammac−/− mice.. J Virol.

[46] Levy JA (1996). The value of primate models for studying human immunodeficiency virus pathogenesis (Review).. Journal of Medical Primatology.

[47] Nathanson N, Hirsch VM, Mathieson BJ (1999). The role of nonhuman primates in the development of an AIDS vaccine (Review).. AIDS.

[48] Warren J (2002). Preclinical AIDS vaccine research: survey of SIV, SHIV, and HIV challenge studies in vaccinated nonhuman primates.. Journal of Medical Primatology.

[49] Naldini L (1998). Lentiviruses as gene transfer agents for delivery to non-dividing cells.. Current Opinion in Biotechnology.

[50] Montini E, Cesana D, Schmidt M, Sanvito F, Ponzoni M (2006). Hematopoietic stem cell gene transfer in a tumor-prone mouse model uncovers low genotoxicity of lentiviral vector integration.. Nat Biotechnol.

[51] Schroder AR, Shinn P, Chen H, Berry C, Ecker JR (2002). HIV-1 integration in the human genome favors active genes and local hotspots.. Cell.

[52] Wu X, Li Y, Crise B, Burgess SM (2003). Transcription start regions in the human genome are favored targets for MLV integration.. Science.

[53] An DS, Qin FX, Auyeung VC, Mao SH, Kung SK (2006). Optimization and functional effects of stable short hairpin RNA expression in primary human lymphocytes via lentiviral vectors.. Molecular Therapy.

[54] Grimm D, Streetz KL, Jopling CL, Storm TA, Pandey K (2006). Fatality in mice due to oversaturation of cellular microRNA/short hairpin RNA pathways.. Nature.

[55] Aagaard L, Zhang J, von Eije KJ, Li H, Saetrom P (2008). Engineering and optimization of the miR-106b cluster for ectopic expression of multiplexed anti-HIV RNAs.. Gene Ther.

[56] Hanawa H, Hematti P, Keyvanfar K, Metzger ME, Krouse A (2004). Efficient gene transfer into rhesus repopulating hematopoietic stem cells using a simian immunodeficiency virus-based lentiviral vector system.. Blood.

